# Supplementary material for: New Facility for Membrane Fouling Investigations under Customizable Hydrodynamics: Validation and Preliminary Experiments with Pulsating Cross-Flow
Source: Membranes (Basel). 2022 Mar 17;12(3):334. doi: 10.3390/membranes12030334 (PMC8951022; doi:10.3390/membranes12030334)
Supplement: Supplementary file 1 [file membranes-12-00334-s001.zip › membranes-1598676-SI.pdf]

New facility for membrane fouling investigations under customisable hydrodynamics: validation and preliminary experiments with pulsating cross-flow

Roberto Bert [1,2,], Costantino Manes [1,2] and Alberto Tiraferri [1,2]

[1] Department of Environmental Land and Infrastructure Engineering (DIATI), Politecnico di Torino, Corso Duca degli Abruzzi, 24 - 10129 Torino, Italy

[2] CleanWaterCenter@PoliTo, Politecnico di Torino, Corso Duca degli Abruzzi, 24 - 10129 Torino, Italy

Supporting Information

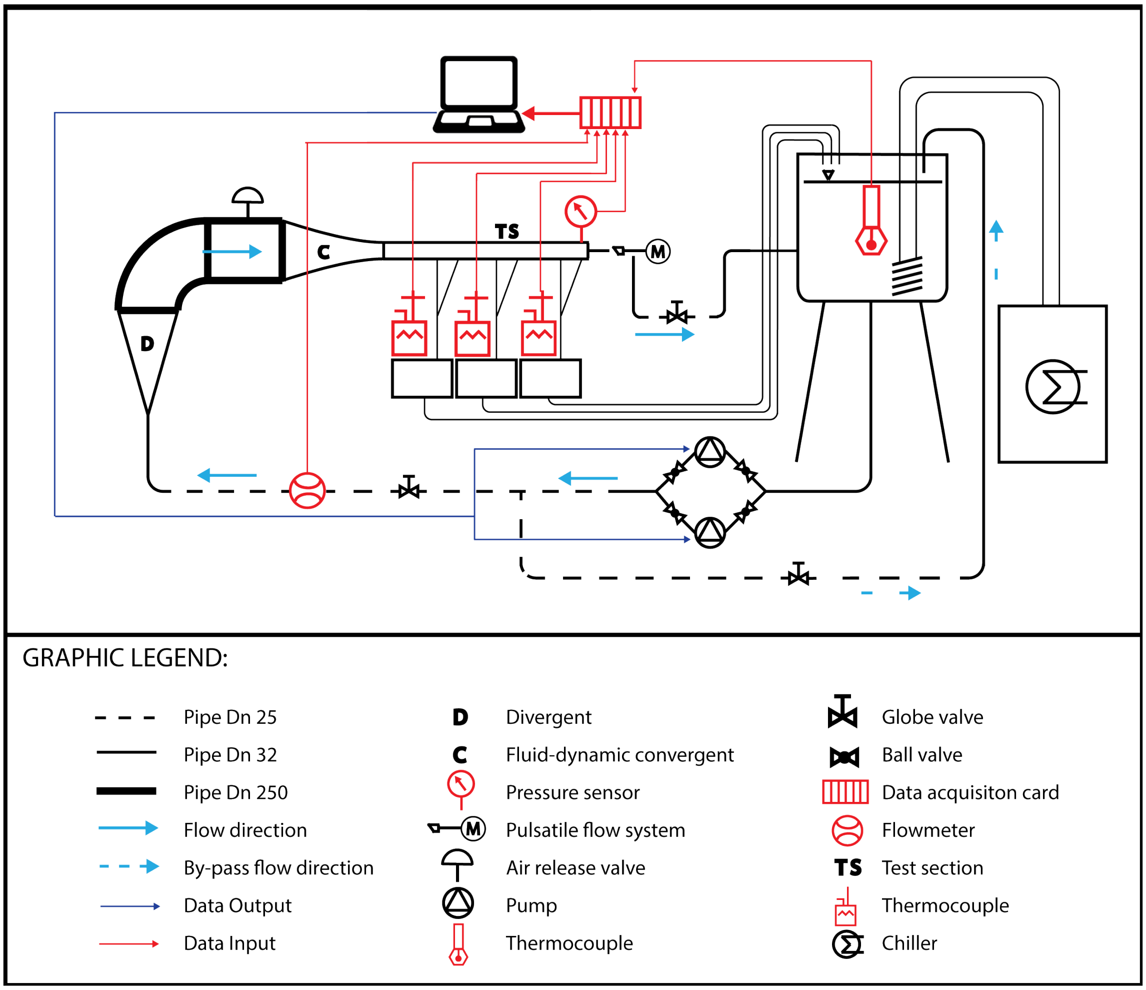

Figure S1: Pipes and instrumentation diagram (i.e. *P&ID* Diagram) of the overall facility.

Table S1: Geometries and properties of the main hydraulic components.

Pipes

DN	PN	ϕ_{est} [mm]	ϕ_{int} [mm]
25	16	26.9	22.9
32	16	42.4	38.4
250	16	273	267

Pumps

Type	Power [kW]	Q_{min} [l/s]	H_{max} [m]	$Q_{70\%}$ [l/s]	$H_{Q_{70\%}}$ [m]	Q_{max} [l/s]	H_{min} [m]
Lowara - 5SV05	0.75	0.67	36.4	1.67	28.2	2.35	17.1
Lowara - 5SV23	4	0.67	168.9	1.67	129.6	2.35	78.2

Tanks

Type	Capacity [l]
Main tank	1000
Collecting tanks	15

Test section

Length L [mm]	Height H [mm]	Width B [mm]	Thickness [mm]
1450	10	200	25

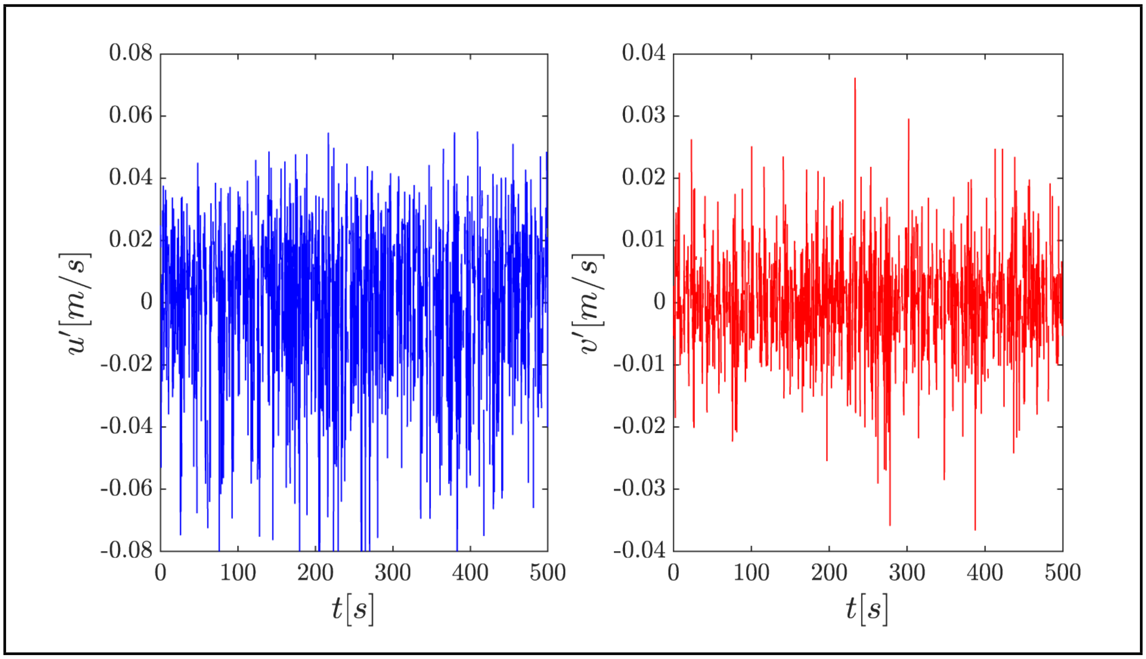

Figure S2: Time series of the longitudinal $u\prime$ and wall-normal $v\prime$ velocity fluctuations deduced by PIV measurement at the position F-2 and coordinates x = 17 mm and y = 2.5.

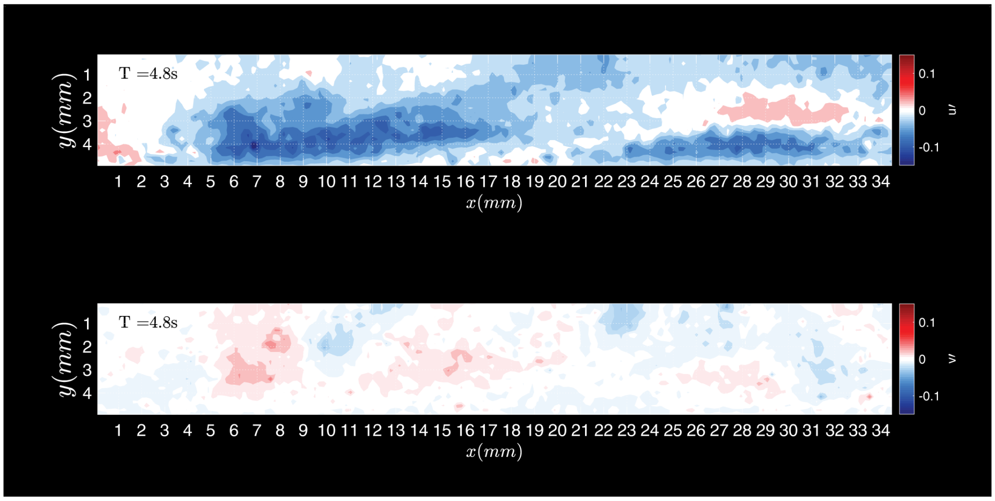

Figure S3: Contour plot of the longitudinal $u\prime$ and wall-normal $v\prime$ velocity fluctuations deduced by PIV measurement at the position F-2 at fixed time T = 4.8 s.

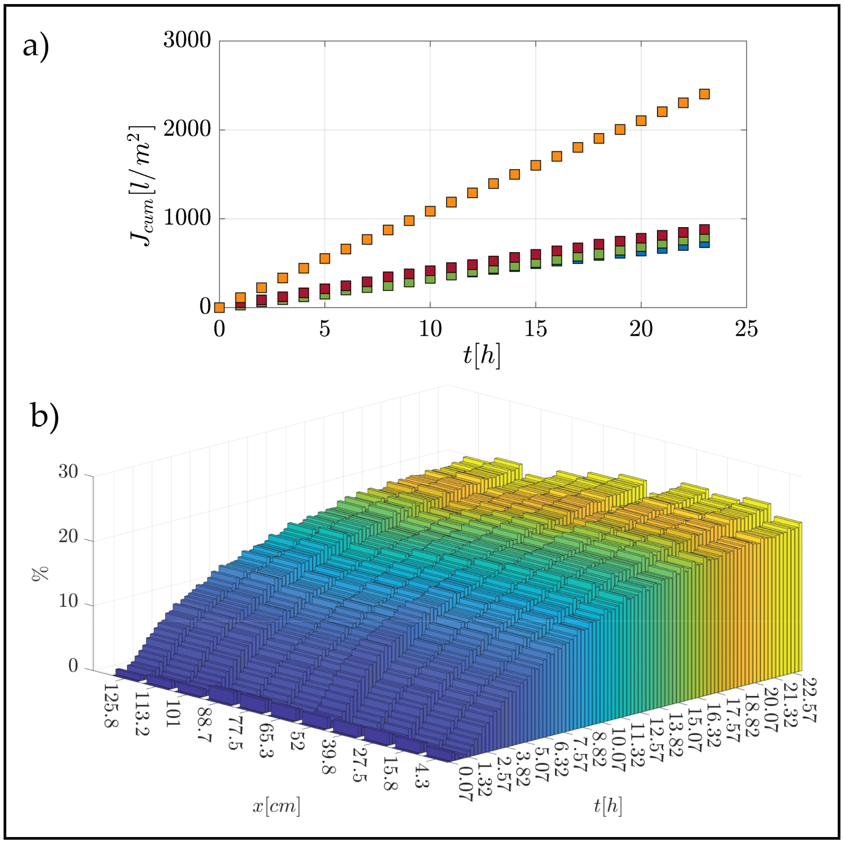

Figure S4: Results of filtration test S - 4.1: (a) Total water flux measured from the individual portions (Where blue, green and red symbols refer to up, mid and down collection tanks respectively and orange squares refer to the cumulative flux) and (b) percentage variation of the membrane colour (yellow band) measured at different longitudinal positions along the membrane surface.
